# Seeking help for sexual difficulties: findings from a study with older adults in four European countries

**DOI:** 10.1007/s10433-019-00536-8

**Published:** 2019-10-11

**Authors:** Sharron Hinchliff, Ana Alexandra Carvalheira, Aleksandar Štulhofer, Erick Janssen, Gert Martin Hald, Bente Træen

**Affiliations:** 1 School of Nursing and Midwifery, University of Sheffield, Sheffield, UK; 2 William James Center for Research, ISPA-University Institute, Lisbon, Portugal; 3 Department of Sociology, University of Zagreb, Zagreb, Croatia; 4 Department of Neurosciences, Institute for Family and Sexuality Studies, University of Leuven, Leuven, Belgium; 5 Department of Public Health, University of Copenhagen, Copenhagen, Denmark; 6 Department of Psychology, University of Oslo, 0317 Oslo, Norway

**Keywords:** Older adults, Sexual difficulties, Seeking help, Sexual well-being, Cross-European

## Abstract

Sexual well-being is an important part of life for many people aged 60 and older. However, older adults often face barriers to seeking and receiving help for sexual difficulties. This study used data from a probability survey (*n* = 3820) on sexuality and ageing to examine help-seeking in 60–75-year-olds in Norway, Denmark, Belgium, and Portugal. More men (12.2%) than women (6.8%) had sought professional help for a sexual difficulty in the past 5 years. The main reason for help-seeking was that sex was important to the participant and/or their relationship. The main source of professional help was the primary care physician. Of those who had sought professional help, 48% were satisfied or very satisfied with the help received, 31.6% were neither satisfied nor dissatisfied, and 20.4% were dissatisfied or very dissatisfied. Others had sought help from informal sources, particularly partners, friends, or websites. The main reasons for not seeking professional help included not being distressed by the symptoms, and thinking that the difficulty would clear up on its own. Multivariable regression analysis revealed that significant correlates of seeking professional help for women and men were level of distress about the sexual difficulty. With regard to women, those who were married, and/or from Portugal were more likely to have sought help. And regarding men, those who attended religious services were more likely to have sought professional help. These results have important implications for healthcare and can be used to inform the development and delivery of services for older adults who experience sexual difficulties.

**Electronic supplementary material:**

The online version of this article (10.1007/s10433-019-00536-8) contains supplementary material, which is available to authorized users.

## Introduction

A substantial body of research indicates that the majority of older adults are sexually active and enjoy a range of sexual activities (DeLamater and Koepsel 2015; Fileborn et al. 2015; Freak-Poli et al. 2017). Sexual activity and intimacy have been found to contribute to the quality of life of older women and men in countries across the world (see Træen et al. 2017a, b). However, sexual engagement and pleasure can be hampered by sexual difficulties, and the likelihood of experiencing a sexual difficulty increases with age (DeLamater 2012). While some older adults seek help for their sexual difficulties, others do not. Currently, little is known about why older adults seek help or what their preferred sources of help are. To advance our knowledge in this area, we examine help-seeking using a large probability sample of women and men aged 60 and older who were recruited across four European countries: Norway, Denmark, Belgium, and Portugal. These countries were selected because of a lack of cross-cultural studies which use comparable data collection strategies and designs, and include identical measures, age ranges, and sociodemographic predictor variables, on the topic within Europe.

### Sexual activity and sexual difficulties

A number of large-scale surveys, conducted predominantly in high income countries, indicate that many older adults engage in sexual activities. For example, the National Survey of Sexual Attitudes and Lifestyles (NATSAL 3) conducted in the UK found that of women aged 65–74 years, 23.1% reported having had vaginal sex with a male partner and 10.3% reported masturbation in the previous month. In the past year, 19.0% of women in this age group had given or received oral sex and 3.6% had had anal sex with a male partner (Mercer et al. 2013). Of men aged 65–74, 37.2% reported vaginal intercourse and 33.1% had engaged in masturbation in the previous month. In the past year, 30.4% had given or received oral sex and 2.9% had had anal sex with a female partner (Mercer et al. 2013). While cultural factors and social mores shape sexual attitudes and behaviours (McLaren 2005), similar results about the sexual activity of older adults have been reported in the USA (Herbenick et al. 2010), Australia (Heywood et al. 2018), the Netherlands (Freak-Poli et al. 2017), and Spain (Palacios-Ceña et al. 2012). These findings indicate similairity in the levels of sexual activity of older adults, potentially on a global scale.

In spite of the variety of sexual activities that older adults engage in, there is consistent evidence that the frequency of sex declines with increase in age (Herbenick et al. 2010; Field et al. 2013; Karraker and DeLamater 2013; Lindau et al. 2007). The reasons for this vary and include the absence of a sexual partner, ill health, and a general “slowing down” with older age (Gott 2004). Older adults tend to adopt a broad definition of sexual activity which includes acts of intimacy which may not be considered sexual by younger people (Minichiello et al. 2004), yet surveys rarely capture such intimacies. And this may connect with the reduced levels of sex reported above.

Both intimacy and sexual activity contribute to well-being and have been identified as quality of life components for older adults who have a sexual partner (Gott and Hinchliff 2003a). In addition to the pleasure associated with consensual and mutually satisfying sex (Berdychevsky and Nimrod 2017; Fileborn et al. 2015; Weeks 2002), there are general benefits to psychological well-being (Berdychevsky and Nimrod 2017) and relationship quality. For example, sexually active older adults tend to report a strong emotional connection with their partner when they consider sex an expression of love and commitment (Berdychevsky and Nimrod 2017; Fileborn et al. 2015; Heiman et al. 2011; Trudel et al. 2010). The American Association of Retired Persons (AARP 2004) found that older adults who reported being in good health and who were satisfied with their sex life also reported a high quality of life. Satisfaction with sex had a more positive effect on men than women with regard to how they rated their quality of life (AARP 2004).

Satisfaction with one’s sexual life can be negatively impacted by the presence of sexual difficulties, some of which are more likely to be experienced with increasing age. These include erection and ejaculation difficulties, decreased sexual desire, and dyspareunia (Træen et al. 2017a). Low levels of sexual activity are associated with poor health (Field et al. 2013; Karraker and DeLamater 2013; Lindau et al. 2007), as health conditions can affect physical sexual function and psychosexual well-being. A number of medications have side-effects which can adversely affect sexual function (e.g. by interfering with desire, arousal, and orgasm), and older adults tend to experience an increased sensitivity to medical drugs, including their side-effects (Bouman 2013).

### Seeking and receiving help

In spite of the importance of sex and intimacy to the well-being of many older adults, there is evidence that they do not always seek professional help when they experience a sexual difficulty (Hinchliff and Gott 2011). Although there is a dearth of empirical data on this topic, the few studies that exist have identified various barriers to help-seeking. Some patients consider their primary care physician a generalist rather than a specialist, and for this reason older adults do not see them as an appropriate source of help for sexual issues (Sarkadi and Rosenqvist 2001). This is an important finding as other studies have identified that the primary care physician would be the first port of call for older adults who wanted help for a sexual difficulty (Gott and Hinchliff 2003b; Tinetti et al. 2018). Other reasons why older adults do not seek help for a sexual difficulty include viewing the problem as a “normal” part of ageing (Moreira et al. 2005; Tinetti et al. 2018), feeling comfortable with how things are, waiting to see if it got better on its own, and not viewing the difficulty as serious (Moreira et al. 2005).

Psychosocial factors also play a role, such as sex being a sensitive, and possibly embarrassing, topic to talk about (Gott and Hinchliff 2003b; Nicolosi et al. 2005, 2006). However, the picture is not straightforward as some studies, for example Farrell and Belza (2012), have found that older women and men *are* comfortable discussing sex with their health providers. Clearly, deciding whether to seek help and from whom is part of a complex process of weighing-up the pros and cons (Azar et al. 2013). Older adults who have sought help for a sexual difficulty report doing so because of the distress it has caused themselves and their partners (Fitter et al. 2009; Hinchliff et al. 2018). However, again, research in this area is limited.

Given the relative paucity of research on this topic, there is a danger that the sexual well-being needs of older adults will continue to be unmet within healthcare. In this article, we use data from our cross-European project on healthy sexual ageing to examine the factors that influence whether older adults seek help for a sexual issue. The findings are important in the context of a growing international sexual rights agenda for older adults (Barrett and Hinchliff 2018). The failure to provide age-friendly services that support sexual well-being runs the risk of preventing older adults from receiving the help they want, which has potential implications for their psychological well-being and romantic relationships. The findings will help to inform healthcare by directing resources to the right places to support healthy sexual ageing.

## Method

### Recruitment, participants, and ethics

National probability-based samples of participants aged 60–75 years were recruited from Norway (*n* = 1271), Denmark (*n* = 1045), Belgium (*n* = 991), and Portugal (*n* = 509). The survey was led by the Department of Psychology at the University of Oslo, in cooperation with Ipsos; a well-established marketing research company. Ipsos conducted recruitment interviews by telephone in each country, where they emphasised that we were interested in the responses of people who were not currently sexually active as well as those who were sexually active. Telephone recruitment was randomized by using national landline and mobile phone registries. However, a different procedure was followed in Portugal because of the lack of such registries, so Ipsos utilised an established method of telephone recruiting. First, telephone numbers were selected at random from phone directories and the Ipsos database of phone numbers. Second, participants were selected by gender and age.

To ensure that the survey was conducted ethically, Ipsos followed national and international rules and guidelines on ethics. The research was guided by the Norwegian Association of Marketing and Opinion Research, and ESOMAR: the European Society for Opinion and Marketing Research (Ipsos is a member of both). Further details about the recruitment, sampling, and data collection can be found in Træen et al. (2018).

### The questionnaire

The questionnaire consisted of 66 items based on the survey’s key domains (see Appended in Electronic Supplementary Material). It was designed in English and translated into native languages by the project team and Ipsos staff based in each country. The design of the questionnaire was based on existing surveys conducted with the target group and thus included key variables identified within those studies. Where possible, we used the original items and only slightly revised the question wording in a few exceptions. The Sexual Relationships and Activities questionnaire (SRA-Q) from the English Longitudinal Study of Ageing provided 13 questions about sexual attitudes (Lee et al. 2016). Indicators of help-seeking were taken from Hinchliff and Gott’s (2011) review paper, and sexual activity, experiences, and sexual functioning came from the British National Survey of Sexual Attitudes and Lifestyles (NATSAL-3) (Mitchell et al. 2013) and Lee et al. (2016) as above. The sociodemographic variables were taken from NATSAL-3 (Mitchell et al. 2013), the Sexual Behavior and Risks of HIV Infection in Europe Survey (Hubert et al. 1998), and the Swedish Sexual Behavior Study 1996 (Lewin et al. 2000).

### Measures

*Help*-*seeking* The participants were asked. “Have you sought professional help for a sexual issue in the last 5 years? Tick all that apply”. The response alternatives were “No”, “Yes, because sexual activity is important to me”, “Yes, because sexual activity is important to our relationship”, “Yes, because my partner wanted me to”, “Yes, because the change in my sex life had a negative impact on how I felt (e.g. sad, depressed, frustrated)”, “Yes, because the change in our sex life had a negative impact on our relationship (e.g. loss of love, tension, argument)”, “Yes, because I feared that my partner would seek another sexual partner”, “Yes, because I was concerned that I would not be able to meet a new sexual partner”, and “Yes, for other reasons”. A new dichotomous variable was created where 1 = “no help-seeking”, and 2 = “Help-seeking”.

*Who help was received from* was tapped by the question “If received professional help, was this from (Tick all that apply)”. The response alternatives were “Primary care physician”, “General practitioner”, “Primary care nurse”, “Secondary care nurse”, “Secondary care doctor”, “Physiotherapist”, “Support worker”, “Social worker”, “Sexual and relationship therapist”, “Psychologist”, and “Other”.

*Satisfaction with help received* was measured by the question “If you received professional help, how satisfied were you with the help you received?” The response categories were 1 = “Have not received help”, 2 = “Very dissatisfied” 3 = “Dissatisfied”, 4 = “Neither dissatisfied nor satisfied”, 5 = “Satisfied”, and 6 = “Very satisfied”.

*Being asked about sexual heath by professionals* was measured by the question “In the last 5 years, has a doctor or other health professional asked you about your sexual life?” The response categories were 1 = “Yes”, and 2 = “No”.

*Reasons for not having sought help* was tapped by the question “If you have experienced a sexual issue and NOT sought help, what was the reason(s) for this? Tick all that apply”. The alternatives were “Embarrassed”, “Ashamed”, “Didn’t have time”, “Expected it would clear up on its own”, “Symptoms did not bother me”, “I did not know where to seek help”, and “Other”.

The predictors of the outcome variables above were: *Sex* (1 = male and 2 = female) and *current age*. *Level of education* was assessed as the highest level of formal education, and recoded into 1 = primary, 2 = secondary, and 3 = tertiary education. *Relationship status*—“Do you currently have a steady/committed relationship with anybody? A steady/committed relationship also includes married/cohabiting persons.” The response categories (1 = yes, 2 = no, and 3 = unsure) were dichotomised into 0 = no or unsure and 1 = in a committed relationship. *Church attendance*—“Apart from special occasions such as weddings, funerals, and baptisms, how often do you attend religious services or meetings?” 1 = once a week or more, 2 = once every 2 weeks, 3 = once a month, 4 = twice a year, 5 = once a year, 6 = less than once a year, and 7 = never. *General health status self*-*assessment*—was measured by the question “In general, would you say your health is:” where the response categories were 1 = excellent, 2 = very good, 3 = good, 4 = fair, and 5 = poor. *Level of distress* about one or more sexual difficulties was indicated by eight items which measured distress related to a particular type of sexual difficulty (e.g. lack of interest in sex, pain during sex, climax reached more rapidly than liked, etc.). The items, which used a 4-point scale ranging from 1 = no distress to 4 = severe distress to anchor answers, were summed into a composite indicator (Cronbach’s alpha was .98 for male and .99 for female participants). Higher composite scores denoted higher distress.

### Data analysis

Apart from the descriptive data analysis, to explore predictors and correlates of seeking professional health for sexual difficulties, multivariable logistic regression analysis (Hidalgo and Goodman 2013) was carried out by gender. Countries were entered in the model as dummy variables, with the largest sample (Norway) acting as reference category. Independent variables were basic sociodemographic characteristics (age, education, relationship status, and religiosity), general self-assessed health status, and level of distress related to the experience of one or more sexual difficulties. The analysis was carried out using unweighted and weighted data in the statistical package SPSS 24. Census-based post hoc weighting by country was employed to correct recruitment-related distortions in age, gender, and region.

## Results

### Characteristics of the samples

Table 1 presents an overview of the four national samples using unweighted and weighted data: the samples are re-weighted for gender, age, and region to match the general population aged 60–75 years in the respective countries.

**Table 1:** Sociodemographic characteristics of samples by country (unweighted and weighted data)

	Unweighted data				Weighted data			
	Norway (%)	Denmark (%)	Belgium (%)	Portugal (%)	Norway (%)	Denmark (%)	Belgium (%)	Portugal (%)
Gender								
Men	53.2	50.7	32.1	46.4	49.9	50.1	49.0	45.6
Women	46.8	49.3	67.9	53.6	50.1	49.9	51.0	54.4
*N*	1270	1045	990	509	1270	1045	990	509
Age group								
60–64	30.9	27.6	33.1	44.8	35.6	27.7	33.5	35.4
65–69	33.3	31.1	36.7	33.0	34.4	31.8	35.6	35.6
70–75	35.9	41.3	30.2	22.2	30.0	40.6	30.9	29.1
*N*	1269	1045	990	509	1272	1045	989	509
Level of education								
Primary	9.9	27.6	13.7	32.9	9.9	27.5	12.2	38.8
Secondary	36.0	37.6	51.5	49.1	37.0	37.6	51.2	44.2
Tertiary	54.0	34.8	34.8	18.0	53.1	34.9	36.6	17.0
*N*	1268	1038	983	505	1270	1045	990	509
Relationship status								
Partnered	76.3	84.2	58.0	83.2	76.4	84.1	63.2	82.5
No partner	23.2	15.8	42.0	16.8	23.6	15.9	36.8	17.5
*N*	1255	1043	985	505	1267	1045	989	509
Church attendance								
Never	34.6	30.5	43.6	23.7	33.8	30.6	44.2	20.7
Less than once a year	23.5	24.0	11.6	15.6	23.4	24.0	12.2	13.8
Once or twice a year	28.0	31.8	24.6	22.3	28.5	31.8	24.2	22.6
Once a month or more often	13.9	13.7	20.2	38.5	14.3	13.6	19.4	42.9
*N*	1257	1036	978	494	1258	1036	981	492
Self-reported health status								
Excellent	12.2	14.2	5.2	3.6	11.8	14.1	5.5	2.7
Very good	30.1	39.8	26.5	11.1	30.4	39.8	26.3	8.7
Good	39.6	28.0	48.7	37.7	39.6	28.1	47.9	35.6
Fair	14.9	16.2	16.9	41.1	15.0	16.2	17.9	44.6
Poor	3.1	1.8	2.8	6.5	3.2	1.8	2.4	8.3
*N*	1191	954	896	506	1270	1045	990	509

### Frequency of help-seeking and related aspects

A small number of participants had sought professional help for a sexual issue in the past 5 years. Of these, more men than women reported that they had sought help: Norwegian men 12%, women 5.2%; Danish men 9.4%, women 5.8%; Belgian men 15.1%, women 8.1%; and Portuguese men 13%, women 9.7%. The main reasons for seeking professional help reported by men (all countries) were: because “sexual activity is important to me”, followed by “sexual activity is important to our relationship”, then “the change in sex life had a negative impact on how I felt”, and “a negative impact on our relationship”. These results were consistent except for Belgium which was lower on the variable “sex is important to our relationship”, and Portugal which was lower on “the change in sex life had a negative impact on our relationship” (Table 2). The main reasons women reported seeking professional help varied more than those reported by the men. The highest responses were: “sex is important to our relationship” (Norway, Denmark); “other reasons” (Belgium, Portugal); closely followed by “sex is important to me” (all countries). Interesting gender differences were noted: Portuguese (11.1%), Norwegian (9.1%) and Danish (3.3%) women reported that they sought help because they “feared my partner would seek another sexual partner” yet out of the men only Norwegians (1.3%) reported this (Table 2). Overall, more men than women had sought help because sex was important to them, and because sex was important to their relationship.

**Table 2:** Reasons for help-seeking for a sexual issue in the last 5 years by country (per cent, weighted data)

	Men				Women			
	Norway (%)	Denmark (%)	Belgium (%)	Portugal (%)	Norway (%)	Denmark (%)	Belgium (%)	Portugal (%)
	*n* = 76	*n* = 49	*n* = 73	*n* = 30	*n* = 32	*n* = 30	*n* = 41	*n* = 27
Because sexual activity is important to me	55.3	57.1	53.4	56.7	21.9	23.3	19.5	22.2
Because sexual activity is important to our relationship	44.7	42.9	14.9	30.0	43.8	25.8	14.6	18.5
Because my partner wanted me to	6.6	10.2	4.1	3.3	12.1	0.0	2.4	3.7
Because the change in my sex life had a negative impact on how I felt	19.7	16.3	15.1	10.0	18.2	6.7	4.9	7.4
Because the change in my sex life had a negative impact on our relationship	13.2	14.6	20.5	10.0	9.4	3.3	12.2	7.4
Because I feared that my partner would seek another sexual partner	1.3	0.0	0.0	0.0	9.1	3.3	0.0	11.1
Because I was concerned that I would not be able to meet a new sexual partner	6.6	6.3	8.2	3.3	3.0	3.3	2.5	0.0
Other reasons	13.2	10.4	11.0	10.0	21.2	23.3	22.0	29.6

Percentages exceed 100%, because multiple answers to the question about reasons for help-seeking were allowed

The main sources of professional help reported by men were the primary care physician (Norway 81.4%; Denmark 87.5%; Belgium 50%) except Portugal which was the secondary care doctor (50%; but closely followed by primary care physician 45.5%). Next, it was the secondary care doctor or nurse, followed by sex and relationship therapist or psychologist, then physiotherapist. For women, it was the primary care physician (Denmark 58.8%; Belgium 56.5%; Portugal 42.9%) except Norway which was the secondary care doctor (54.2%; this was closely followed by primary care physician 41.7%), followed by secondary care doctor, and then sex and relationship therapist or psychologist. Overall, for women and men, the level of satisfaction with professional help received was “very satisfied” (9.2%), “satisfied” (38.8%), “neither dissatisfied nor satisfied” (31.6%), “dissatisfied” (12.7%), and “very dissatisfied” (7.7%). The majority of women and men in all four countries had *not* been asked about their sex life by a doctor/health professional in the past 5 years: this ranged from 73.2% (Portuguese women) to 89.6% (Belgian men).

With regard to those who had experienced a sexual issue but had not sought professional help, the main reasons reported by men were: because “the symptoms did not bother me” (12.8% Norway; 14.9% Denmark; 18.1% Belgium; 9.6% Portugal), “expected it would clear up on its own” (8.6% Norway; 12% Denmark; 11.5% Belgium; 14.5% Portugal), and “other”. A similar pattern was observed for women. The main reasons they had not sought professional help were: because “the symptoms did not bother me” (15.9% Norway; 14.7% Denmark; 18.1% Belgium; 21.4% Portugal), “expected it would clear up on its own” (7.5% Norway; 10.8% Denmark; 10.1% Belgium; 5.0% Portugal), and “other”. Another main reason for not seeking help for women and men was embarrassment (Table 3).

**Table 3:** Reasons for not seeking help for a sexual issue among participants who had at least one sexual problem, by country (per cent, weighted data)

	Men				Women			
	Norway (%)	Denmark (%)	Belgium (%)	Portugal (%)	Norway (%)	Denmark (%)	Belgium (%)	Portugal (%)
	(*n* = 443)	(*n* = 368)	(*n* = 374)	(*n* = 166)	(*n* = 440)	(*n* = 334)	(*n* = 326)	(*n* = 160)
Embarrassed	5.2 (23)	5.2 (19)	7.2 (27)	3.0 (5)	2.7 (12)	4.8 (16)	8.9 (29)	4.4 (7)
Ashamed	0.9 (4)	0.5 (2)	6.4 (24)	4.8 (8)	0.5 (2)	0.6 (2)	5.5 (18)	3.1 (5)
Did not have time	0.5 (2)	0.8 (3)	1.3 (5)	0.6 (1)	0.0 (0)	0.6 (2)	1.5 (5)	0.0 (0)
Expected it would clear up on its own	8.6 (38)	12.0 (44)	11.5 (43)	14.5 (24)	7.5 (33)	10.8 (36)	10.1 (33)	5.0 (8)
Symptoms did not bother me	12.8 (57)	14.9 (55)	18.1 (68)	9.6 (16)	15.9 (70)	14.7 (49)	18.1 (59)	21.4 (34)
I did not know where to seek help	1.6 (7)	2.2 (8)	4.3 (16)	3.0 (5)	0.7 (3)	2.1 (7)	1.2 (4)	1.9 (3)
Other	11.3 (50)	16.0 (59)	16.6 (62)	9.6 (16)	11.1 (49)	17.7 (59)	16.8 (55)	9.4 (15)

Those who answered that they had *not* sought professional help in the last 5 years were asked “Have you sought help for a sexuality related issue or problem from any of the following sources in the past 5 years?”. The highest response for both men and women was that they had talked to their partner (all countries). Women’s second main source of help was their friends and men’s was through using the Internet/websites.

### Correlates of professional help-seeking for sexual difficulties

Two separate multivariable logistic regressions were carried out among women and men (Table 4) who reported at least one sexual difficulty and therefore answered the questions about levels of distress. Portuguese women were about 2.40 times more likely to have sought professional help than Belgian and Danish women. Women with tertiary education level were 2.40 times more likely to have sought professional help than were women with a lower educational level. Relationship status was positively associated with help-seeking in women but not men. Married women were 2.32 times more likely to have sought professional help than non-committed women. Moreover, women who reported a higher level of distress over sexual difficulties were 1.14 times more likely to have sought professional help than women who reported a lower level of distress over sexual difficulties. General health was also a significant correlate of professional help-seeking.

**Table 4:** Correlates of seeking professional help for sexual difficulties by gender

	Women		Men	
	AOR^a^	AOR^a^	AOR^a^	AOR^a^
	(95% CI)	(95% CI)	(95% CI)	(95% CI)
Denmark dummy	1.38	1.05	.59*	.62*
	(.66–2.90)	(.55–2.00)	(.37–.94)	(.44–1.08)
Belgium dummy	1.65	1.41	.87	.69
	(.83–3.29)	(.80–2.48)	(.58–1.32)	(.44–1.08)
Portugal dummy	2.54*	2.40*	.85	.73
	(1.11–5.80)	(1.13–5.09)	(.48–1.50)	(.44–1.22)
Age	1.00	1.00	1.03	1.02
	(.94–1.06)	(.95–1.06)	(.99–.1.07)	(.99–1.06)
Education				
Primary^b^	1	1	1	1
Secondary	1.91*	1.53	.92	.91
	(1.07–3.42)	(.93–2.53)	(.63–1.33)	(.64–1.30)
Tertiary	3.00*	2.40*	.71	.57*
	(1.21–7.40)	(1.13–5.09)	(.42–1.19)	(.34–.97)
In a relationship/married	3.16**	2.32**	1.09	1.09
	(1.44–6.94)	(1.26–4.27)	(.68–1.75)	(.69–1.73)
Settlement size	.85	.88	.99	1.03
	(.69–1.04)	(.73–1.05)	(.86–1.14)	(.91–1.18)
Religiosity	1.05	1.02	1.10*	1.09*
	(.92–1.20)	(.91–1.15)	(1.01–1.21)	(1.00–1.19)
General health	1.61**	1.34*	1.05	1.05
	(1.20–2.16)	(1.06–1.76)	(.88–1.25)	(.89–1.25)
Level of distress over one or more sexual difficulties	1.13***	1.14***	1.14**	1.15***
	(1.09–1.18)	(1.10–1.19)	(1.11–1.18)	(1.11–1.18)

^a^Adjusted odds ratio

^b^Referent category

*p < .05; **p < .01; ***p < .00

Among men, educational level, religiosity, and level of distress over sexual difficulties were associated with professional help-seeking. Men with a higher level of attendance to religious services were 1.09 times more likely to have sought professional help than were men with a lower level of attendance to religious services. Men who reported a higher level of distress over sexual difficulties were 1.15 times more likely to have sought professional help than men who reported a lower level of distress over sexual difficulties.

When the analyses were repeated using weighted data, the pattern of findings remained largely the same. The only two exceptions were the finding that secondary education, contrasted to primary education, ceased to be a significant predictor of help-seeking in women and that college educated men, in contrast to those with only primary education, had 43% lower odds of help-seeking.

## Discussion

This quantitative study examined healthy sexual ageing in a large cross-European sample of adults aged 60 and older. Our findings support those of earlier studies (Hinchliff et al. 2018) that when sex is important to the participant and/or their relationship they will seek professional help when they experience a sexual difficulty. Indeed, our regression analyses confirmed that distress about a sexual issue was a key driver of seeking professional help in both women and men. The questions which asked whether participants had sought professional help in the last 5 years because of the negative impact on themselves or their relationship, specifically led them to consider psychological components (sad, depressed, frustrated; loss of love, tension, arguments) which are indicators of distress.

Sexual activity, as an important component of the romantic relationship, and sexual difficulties as having a negative impact on the individual and their relationship, were key reasons why participants sought professional help. This is consistent with the findings of other studies (e.g. Fitter et al. 2009; Hinchliff et al. 2018) and gives an indication of the place and meaning of sex within the lives of some older adults. Distress about sex was a significant correlate of seeking professional help for women and men across all countries. Women who were Portuguese, or had tertiary education, or were married, were more likely to have sought professional help. And for men, educational level and religiosity were significant correlates of professional help-seeking. It may be that Portuguese women are more likely to report seeking professional help because a high number of clinicians are trained in sexual medicine in Portugal, and the women therefore had access to such help.

Our findings are consistent with previous research which has found that the main source of professional help for older women and men is the primary care physician (Gott and Hinchliff 2003b). The next key source of professional help in the present study was the secondary care doctor. Taken together, these findings indicate that participants who sought professional help are likely to have considered their sexual difficulty to be a medical one. Sexual communication between patients and healthcare professionals is, however, far from straightforward, and studies have identified that sex can be a difficult topic to talk about in healthcare settings by patients and professionals alike. Patient barriers include embarrassment and fear of being judged negatively for showing an interest in sex at their age (see Gott 2004). Some participants in the present study mentioned embarrassment as a reason for not seeking professional help for a sexual issue. Professional barriers include embarrassment, lack of consultation time, and not wanting to invade the patient’s privacy (Hinchliff and Gott 2011). Again, our findings lend support here as they show that only a minority of participants (overall 14.5% of men and 14.7% of women) had been asked about their sexual life by a doctor or other health professional in the last 5 years.

Of those who had sought professional help, satisfaction with the help received varied. Around one-third of the sample was neither satisfied nor dissatisfied, and one-fifth was dissatisfied or very dissatisfied. The limited qualitative research that has been conducted on this topic may provide insight into the reasons for dissatisfaction found in the present study. Participants in those studies reported discriminatory practices which they believed to be acts of ageism by the doctor. These included being referred to other sources (e.g. psychological services) which participants believed to be inappropriate and a way that the doctor could avoid dealing with the patient and their concerns (Fitter et al. 2009). Empirical data show that older patients who seek help for a sexual issue are satisfied when they feel they have been listened to and that their sexual concerns have been taken seriously (Hinchliff et al. 2018). The dissatisfaction reported in this body of research as a whole indicates that more needs to be done to ensure that the sexual well-being needs of older patients are met in a satisfactory way.

The main reasons for not seeking professional help in the present study included not being bothered by the symptoms, or thinking that the issue would clear up on its own. Again, these findings echo those of other studies on the sexual well-being of older adults, in particular the Global Survey of Sexual Attitudes and Behaviors (see Moreira et al. 2005). Not being bothered by the symptoms could be explained by the findings reported by DeLamater and Koepsel (2015) that sex becomes less important than the “emotional intimacy, stability, and continuity” of a relationship in older age. Some participants in the present study had not sought professional help but had spoken with their partner, friends, or used the Internet. The Internet as a source of information for sexual health and well-being in older adults is consistent with research conducted by Fileborn et al. (2017) whose qualitative study of older Australians found that the Internet was the main source of information for those who required sexual education or support (e.g. on topics such as sexually transmitted infections, and to purchase sex toys or condoms). In the present study, men were more likely to use the Internet in this way, whereas women were more likely to speak with friends. The latter was also found in the Global Study of Sexual Attitudes and Behaviors (Moreira et al. 2008) and may be explained by gender-role socialisation where, from a young age, girls are encouraged to engage socially with other girls and to continue this throughout adulthood (Coupland 2014).

More men than women in the present study had sought professional help for a sexual issue in the last 5 years. This may be explained, as Loe (2004) describes, by the widespread awareness of Viagra bringing male sexual problems into the public consciousness, and thus making them easier (for men) to talk about. The main reason men (across all countries) gave for seeking professional help was because sex was important to them, whereas a main reason for women was because sex was important to their relationship. Indeed, relationship status was positively associated with help-seeking in women but not men. Again, this could reflect gender-role socialisation in that heterosexual women are encouraged to maintain harmony within their relationship (Stoppard and McMullen 2003), and one way to do this is to put the relationship first. Similarly, women from three countries, compared to (a very small percentage of) men from one country cited a reason for seeking professional help as fear of their partner seeking another sexual partner. It is possible that this reflects the male sexual drive discourse that was popular in Europe during their upbringing, and which may still influence their beliefs today. Women from Portugal and Norway were more likely to report this, with little difference in numbers between them, which is interesting because Portugal is associated with traditional gender relations and Norway with gender equality (Lewin et al. 2000). It perhaps demonstrates, therefore, the pervasiveness of the heterosexual male sexual drive discourse. Seeking help because sex was important to the relationship was higher in Norway and Denmark for men and women, which again might reflect that Nordic countries are recognised for greater gender equality.

As indicated above, sexual difficulties can be a source of distress by having a negative impact on the relationships as well as psychological well-being. Thus, there is a clear need to provide sexual well-being support for older adults who require it. This requirement is particularly prominent in the context of an increasing global population of older adults, many of whom are likely to experience a long-term health condition where the condition itself and the medications prescribed to manage it can affect sexual function. The primary care physician was the main source of professional help for participants in the present study, and thus, primary care organisations can be utilised for sexual well-being support. Barrett and Hinchliff (2018) describe a framework for achieving the sexual rights of older adults which service-providers can tailor to their own needs and user populations. The main recommendation for healthcare practice is to have a sexual rights policy for older adults in place, as this will raise awareness of the rights and help to prevent age-related discriminatory practice. A further step would be to provide information about sexual health and well-being for health professionals and patients. There is a significant lack of educational resources for older adults around sexual health, sexual well-being, and intimate relationships (Barrett and Hinchliff 2018). Older adults have specially requested more information about the “normal” changes to sexual well-being with age (Hinchliff and Gott 2004; Fileborn et al. 2015).

While this study has strengths, in particular that identical measures and comparable data collection were employed across all four countries, there are limitations too. These include the low response rate from some countries and lack of sexual diversity overall: only a small number of sexual minorities took part in the study which prevents meaningful analysis of factors associated with sexual help-seeking for diverse sexual orientations. Unfortunately, we did not collect data on ethnicity, or those who declined to participate and the reasons why they declined. The majority of participants were well-educated (most had received secondary or tertiary level education) and were sexually active which may have influenced their decision to take part in the study. There is potential that only older adults who hold liberal views around sexual activity took part in the study, although the questionnaire was completed anonymously which is known to encourage participation in sex-related research (Ericksen and Steffen 1999). The findings are relevant to community dwelling older adults, so we need to ask if help-seeking would look different in those who lived in care or residential homes. Indeed, the cut-off age of 75 years means that we do not know anything about help-seeking of those aged older than 75. These factors affect the transferability of the findings beyond the current project. Further research is therefore required which examines other populations, and those less likely to take part in sex-research, if we are to build the evidence base on help-seeking for sexual difficulties.

## Conclusion

The findings of this study provide insights into the reasons why older adults do or do not seek help for sexual issues. Distress about a sexual issue is linked with seeking professional help, and the primary care physician is the main source of help. However, it may be that the barriers for seeking help are more significant than expected as there were high rates of sexual difficulties in our study (Hald et al. 2019), but only a small number of participants had sought help. The findings can help to direct resources to the right places to support the sexual needs of older adults, which is important in the context of an increasing global population of adults aged 60+ and a growing international sexual rights agenda for older adults.

## Electronic supplementary material

Below is the link to the electronic supplementary material.

Supplementary material 1 (PDF 116 kb)
