# Supplementary material for: Seeking help for sexual difficulties: findings from a study with older adults in four European countries
Source: Eur J Ageing. 2019 Oct 11;17(2):185–95. doi: 10.1007/s10433-019-00536-8 (PMC7292843; doi:10.1007/s10433-019-00536-8)
Supplement: Supplementary file 1 — Supplementary material 1 (PDF 116 kb) [file 10433_2019_536_MOESM1_ESM.pdf]

Questionnaire - sexual health and sexual habits among adults 60-75 years

**Project**	**1600199801**
**Schema ID**	

For each question, read the question text and answers carefully before you tick your answer in the box to the right or below the answer that fits best. This questionnaire should be completed on your own and without sharing your answers with others. Your answers will be treated confidentially, and no one can know who has answered what.

1 What is your gender?

Male ☐ 1
Female ☐ 2
Other ☐ 3

2 What year were you born?

Write your year of birth: ☐☐☐☐ 1

3 What is your highest level of formal education?

One answer only

Primary school (6-8 years at school) ☐ 1
Lower secondary school (9-10 years at school) ☐ 2
Higher secondary school, high school (12-13 years at school) ☐ 3
College, lower university level (Bachelor degree level or similar) ☐ 4
Higher university level (Master degree, PhD. level or similar) ☐ 5

4 Do you currently have a steady/ committed relationship with anybody? A steady/ committed relationship also includes married/ cohabiting persons.

One answer only. Follow instruction to next question.

Yes (⇒ 8) ☐ 1
No (⇒ 5) ☐ 2
Unsure (⇒ 5) ☐ 3

5 Have you ever had a steady/ committed relationship?

One answer only. Follow instruction to next question.

Yes (⇒ 6) ☐ 1
No (⇒ 12) ☐ 2
Unsure, don't wish to say (⇒ 12) ☐ 3

6 If had a steady/ committed relationship:

How many years did the last steady/ committed relationship last?

Write number of years (if less than 6 months, write 00, if 6-12 months, write 01): ☐☐ 1

Write your answer, go to the next question

7 If had a steady/ committed relationship:

How many years ago did the last steady/ committed relationship end?

Write number of years (if less than 6 months, write 00, if 6-12 months, write 01): ☐☐ 1

Write your answer, skip to question 12

8 If you have a steady/ committed relationship:

How long have you been in this steady/ committed relationship?

Write number of years (if less than 6 months, write 00, if 6-12 months, write 01): ☐☐ 1

9 If you have a steady/ committed relationship:

Is your steady/ committed relationship with a man or a woman?

- Man ☐ 1
- Woman ☐ 2
- Other ☐ 3

10 If you have a steady/ committed relationship:

How old is your partner?

Write the age of your partner: ☐

11 If you have a steady/ committed relationship:

Is your partner currently . . .

Tick all that apply

- In paid work/employed ☐ 1,
- Retired from paid work ☐ 2,
- Doing unpaid or voluntary work ☐ 3,
- Looking after home or family, unpaid ☐ 4,
- Other ☐ 5.

12 **FOR ALL:**

How would you describe the town or community where you live?

One answer only

- Rural (under 5,000 inhabitants) ☐ 1
- Small town (5,001-50,000 inhabitants) ☐ 2
- Medium-sized city (from 50,001-200,000 inhabitants) ☐ 3
- Suburb of a large-sized city (more than 200,000 inhabitants) ☐ 4
- Downtown or in the central district of a large-sized city (more than 200,000 inhabitants) ☐ 5
- Other ☐ 6

13 **Are you currently.............?**

Tick all that apply

- In paid work/employed ☐ 1,
- Retired from paid work ☐ 2,
- Doing unpaid or voluntary work ☐ 3,
- Looking after home or family, unpaid ☐ 4,
- Other ☐ 5.

14 **Which of the options below describes best how you currently think of yourself?**

One answer only

- Heterosexual ☐ 1
- Gay/ Lesbian ☐ 2
- Bisexual ☐ 3
- Other ☐ 4

15 **Do you currently regard yourself as belonging to any particular religion?**

One answer only

- No ☐ 01
- Yes, Christian - no particular denomination ☐ 02
- Yes, Roman Catholic ☐ 03
- Yes, Protestant/Church of England/Anglican ☐ 04
- Yes, Free Church/Non conformist/ Evangelical ☐ 05
- Yes, Hindu ☐ 06
- Yes, Jew ☐ 07
- Yes, Islam/Muslim ☐ 08
- Yes, Buddhist ☐ 09
- Yes, Other ☐ 10
- Don't know ☐ 11

16 **Apart from special occasions such as weddings, funerals and baptisms, how often do you attend services or meetings connected with your religion?**

One answer only

- Never ☐ 1
- Less than once a year ☐ 2
- Once a year ☐ 3
- Twice a year ☐ 4
- Once a month ☐ 5
- Once every two weeks ☐ 6
- Once a week or more ☐ 7

THE FOLLOWING QUESTIONS ASK ABOUT YOUR HEALTH AND QUALITY OF LIFE

17 **Has a doctor ever told you that you have any of the medical conditions listed below?**

Tick all that apply

- Arthritis ☐ 01,
- Heart attack ☐ 02,
- Coronary Artery disease / angina / other form of heart disease ☐ 03,
- Hypertension, also known as high blood pressure ☐ 04,
- Stroke ☐ 05,
- Diabetes, also known as high blood sugar ☐ 06,
- Chronic Lung disease (not including asthma) ☐ 07,
- Parkinson's disease ☐ 08,
- Epilepsy ☐ 09,
- **If you are a man:** Benign prostatic hypertrophy ☐ 10,
- **If you are a man:** Prostate cancer ☐ 11,
- **If you are a woman:** Cancer in the uterus or ovaries ☐ 12,
- **If you are a woman:** Breast cancer ☐ 13.

18 **Are you currently taking any type of medicine prescribed by a doctor for depression?**

One answer only

- Yes ☐ 1
- No ☐ 2

19 If you are a woman: **Have you ever taken hormone replacement therapy (HRT)?**

One answer only

☆

- Yes ☐ 1
- No ☐ 2

To all: Now some questions about your views on your health. If you are unsure how to answer a question, please give the best answer you can.

20 **In general, would you say your health is:**

One answer only

- Excellent ☐ 1
- Very good ☐ 2
- Good ☐ 3
- Fair ☐ 4
- Poor ☐ 5

21 The following questions are about activities you might do during a typical day.

Does your health now limit you in these activities? If so, how much?

Tick one box on each line

	A lot (1)	To some extent (2)	A little (3)	Not at all (4)	
• Moderate activities such as moving a table, pushing a vacuum cleaner, bowling, or playing golf?	☐	☐	☐	☐	1
• Climbing several flights of stairs?	☐	☐	☐	☐	2

22 **During the past 4 weeks , have you had any of the following problems with your work or other regular daily activities as a result of your physical health ?**

Tick one box on each line

	Yes (1)	No (2)	
• Accomplished less than you would like?	☐	☐	1
• Been limited in the kind of work or other activities you do?	☐	☐	2

23 **During the past 4 weeks , have you had any of the following problems with your work or other regular daily activities as a result of any emotional problems (such as feeling depressed or anxious)?**

Tick one box on each line

	Yes (1)	No (2)	
• Accomplished less than you would like?	☐	☐	1
• Did work or activities less carefully than usual?	☐	☐	2

24 **During the past 4 weeks, how much did pain interfere with your normal work (including work outside the home and housework)?**

One answer only

- Not at all ☐ 1
- A little bit ☐ 2
- Moderately ☐ 3
- Quite a bit ☐ 4
- Extremely ☐ 5

25 These questions are about how you have been feeling during the past 4 weeks. For each question, please give the one answer that comes closest to the way you have been feeling.

How much of the time during the past 4 weeks . . .

Tick one box on each line

	All the time 1	Most of the time 2	A good bit of the time 3	Some of the time 4	A little of the time 5	None of the time 6	
• Have you felt calm and peaceful?	☐	☐	☐	☐	☐	☐	1
• Did you have a lot of energy?	☐	☐	☐	☐	☐	☐	2
• Have you felt down-hearted and blue?	☐	☐	☐	☐	☐	☐	3

26 **During the past 4 weeks, how much of the time has your physical health or emotional problems interfered with your social activities (like visiting friends, relatives, etc.)?**

One answer only

- All the time ☐ 1
- Most of the time ☐ 2
- A good bit of the time ☐ 3
- Some of the time ☐ 4
- A little of the time ☐ 5
- None of the time ☐ 6

27 **How much do you weigh at present?**

Please record in kilograms: ☐☐☐ 1

28 **How tall are you?**

Please record in centimeters: ☐☐☐ 1

29 **How many hours per week do you exercise?**

Apart from sports please also include household and gardening activities, walks, and bike rides to and from work and/or leisure activities

One answer only

- None ☐ 1
- About ½ hour per week ☐ 2
- About 1-2 hours per week ☐ 3
- About 3-4 hours per week ☐ 4
- About 5-6 hours per week ☐ 5
- 7+ hours per week ☐ 6

30 **On average, how much do you usually smoke during a typical week?**

Write number of cigarettes/cigars/cheroots or cigarillos

If you don't smoke, write 000

. ☐☐☐ 1

31 **On average, how many alcoholic beverages do you consume in a typical week?**

Write the average number of glasses in each line. If you don't drink the kind of beverage, write 00.

- Number of glasses of beer? ☐☐ 1
- Number of glasses of wine? ☐☐ 2
- Number of glasses of Port wine, Sherry etc.? ☐☐ 3
- Number of glasses of spirits? ☐☐ 4

32 **Below there are some statements with which you may agree or disagree. Using the scale below, please indicate your agreement with each item by ticking your answer below the appropriate alternative. Please be open and honest in your responding**

Tick one box on each line

	Strongly agree 1	Agree 2	Neither agree nor disagree 3	Disagree 4	Strongly disagree 5	
• In most ways my life is close to my ideal	☐	☐	☐	☐	☐	1
• The conditions of my life are excellent	☐	☐	☐	☐	☐	2
• So far I have gotten the important things I want in life	☐	☐	☐	☐	☐	3
• I am satisfied with my life	☐	☐	☐	☐	☐	4
• If I could live my life over, I would change almost nothing	☐	☐	☐	☐	☐	5
• I am satisfied with my physical appearance	☐	☐	☐	☐	☐	6
• I don't like the way I look	☐	☐	☐	☐	☐	7
• The way I look makes me feel good about myself ..	☐	☐	☐	☐	☐	8
• The way I look makes me unattractive	☐	☐	☐	☐	☐	9
• My body and face look pretty much the way I would like	☐	☐	☐	☐	☐	10
• I feel bad about my body and my appearance	☐	☐	☐	☐	☐	11
• For me my appearance is an important part of who I am	☐	☐	☐	☐	☐	12
• I am often aware of the way that I look to other people	☐	☐	☐	☐	☐	13
• In most situations, I find myself aware of the way my face and body look	☐	☐	☐	☐	☐	14
• I often think about the impression that the appearance of my face and body make	☐	☐	☐	☐	☐	15
• I am usually conscious of my appearance	☐	☐	☐	☐	☐	16

33 **During the past 4 weeks , how much were you bothered by............**

Tick one box on each line

	Not at all 1	A little 2	Moderately 3	Quite a bit 4	Extremely 5	
• Feeling blue	☐	☐	☐	☐	☐	1
• Feeling suddenly scared for no reason	☐	☐	☐	☐	☐	2
• Feelings of worthlessness	☐	☐	☐	☐	☐	3
• Nervousness or shakiness inside	☐	☐	☐	☐	☐	4
• Thoughts about ending your life	☐	☐	☐	☐	☐	5
• Spells of terror or panic	☐	☐	☐	☐	☐	6
• Feelings of being trapped or caught	☐	☐	☐	☐	☐	7
• Worrying too much	☐	☐	☐	☐	☐	8
• Feeling lonely	☐	☐	☐	☐	☐	9
• Blaming yourself for things	☐	☐	☐	☐	☐	10

34 **The next three questions are about how you feel about different aspects of your life. For each one, please indicate how often you feel that way.**

Tick one box on each line

	Hardly ever 1	Some of the time 2	Often 3	
• How often do you feel that you lack companionship?	☐	☐	☐	1
• How often do you feel left out?	☐	☐	☐	2
• How often do you feel isolated from others?	☐	☐	☐	3

THE FOLLOWING QUESTIONS ARE ABOUT YOUR ATTITUDES AND BELIEFS

35 **This question asks about your personal attitudes to sex. Please tick the one response that best describes your reaction to each of the following statements.**

Tick one box on each line

	Strongly agree 1	Agree 2	Neither agree nor disagree 3	Disagree 4	Strongly disagree 5	
• Having sexual relations before marriage is wrong	☐	☐	☐	☐	☐	1
• A married person having sexual relations with someone other than their spouse is wrong	☐	☐	☐	☐	☐	2
• Two adults of the same sex having sexual relations is wrong	☐	☐	☐	☐	☐	3
• Having one night stands is wrong	☐	☐	☐	☐	☐	4
• Satisfactory sexual relations are essential to the maintenance of a long-term relationship	☐	☐	☐	☐	☐	5
• Sexual relations without love is OK	☐	☐	☐	☐	☐	6
• People are under a lot of pressure to have sex nowadays	☐	☐	☐	☐	☐	7
• There's too much sex in the media nowadays	☐	☐	☐	☐	☐	8
• Young people today start having sex too early	☐	☐	☐	☐	☐	9
• Men have a naturally higher sex drive than women	☐	☐	☐	☐	☐	10
• The ability to have sex decreases as a person grows older	☐	☐	☐	☐	☐	11
• Sexual changes that occur with age are not important to older people	☐	☐	☐	☐	☐	12
• Being sexually active is physically and psychologically beneficial to older people	☐	☐	☐	☐	☐	13

WE WILL NOW ASK YOU SOM QUESTIONS RELATED TO YOUR SEX LIFE AND SEXUAL EXPERIENCES

The questions that follow use terms like sexual intercourse, oral sex and vaginal intercourse. So that everyone attaches the same meaning to these terms, they are explained:

PARTNERS OR SEXUAL PARTNERS: People who have had sex together - whether just once, or a few times, or as regular partners, or as married partners.

VAGINAL SEX (vaginal sexual intercourse): A penis, or other objects inserted in a woman's vagina.
ORAL SEX (oral sexual intercourse): A woman's/ man's mouth on a partner's genital area.
ANAL SEX (anal sexual intercourse): A penis or other objects inserted in own/partner's anus (rectum or back passage).
SEXUAL INTERCOURSE: This includes vaginal, oral and/or anal sex with another person.
HAVING/ HAD SEX: This include sexual intercourse, masturbation, petting or fondling.

36 **Have you ever in your lifetime had any sexual activity (sexual intercourse, masturbation, petting or fondling)?**

One answer only

- Yes ☐ 1
- No ☐ 2

37 **What activities related to love and sexuality do you engage in on the Internet?**

Tick all that apply

- I am not doing anything related to love and sexuality on Internet ☐ 01,
- Using social media such as Facebook or Twitter to find a committed partner ☐ 02,
- Checking out dating sites to find a committed partner ☐ 03,
- Using social media such as Facebook or Twitter to find a partner for sex ☐ 04,
- Checking out dating sites to find sex partners ☐ 05,
- Reading or watching erotica or pornography ☐ 06,
- Seeking sexuality counseling ☐ 07,
- Seeking information or advice ☐ 08,
- Buying sex products (sex-toys, erotica, Viagra, lubricants etc.) ☐ 09,
- Other ☐ 10.

38 **Compared to 10 years ago, how would you rate your interest in sex?**

One answer only

- Much lower ☐ 1
- Somewhat lower ☐ 2
- About the same ☐ 3
- Somewhat higher ☐ 4
- Much higher ☐ 5

39 **How satisfied are you with the current level of sexual activity in your life, in a general way?**

One answer only

- Very satisfied ☐ 1
- Rather satisfied ☐ 2
- Neither satisfied nor dissatisfied ☐ 3
- Rather dissatisfied ☐ 4
- Very dissatisfied ☐ 5

40 **Have you sought professional help for a sexual issue in the last 5 years?**

Tick all that apply

- No **(⇒ 43)** ☐ 1,
- Yes, because sexual activity is important to me ☐ 2,
- Yes, because sexual activity is important to our relationship ☐ 3,
- Yes, because my partner wanted me to ☐ 4,
- Yes, because the change in my sex life had a negative impact on how I felt (e.g. sad, depressed, frustrated) ☐ 5,
- Yes, because the change in our sex life had a negative impact on our relationship (e.g. loss of love, tension, arguments) ☐ 6,
- Yes, because I feared that my partner would seek another sexual partner ☐ 7,
- Yes, because I was concerned that I would not be able to meet a new sexual partner ☐ 8,
- Yes, for other reasons ☐ 9.

41 **If received professional help, was this from:**

Tick all that apply

☆

- Primary care physician / General practitioner ☐ 01,
- Primary care nurse ☐ 02,
- Secondary care nurse ☐ 03,
- Secondary care doctor ☐ 04,
- Physiotherapist ☐ 05,
- Support worker ☐ 06,
- Social worker ☐ 07,
- Sexual and relationship therapist ☐ 08,
- Psychologist ☐ 09,
- Other ☐ 10.

42 **If you received professional help, how satisfied were you with the help you received?**

One answer only

☆

- Very dissatisfied ☐ 1
- Dissatisfied ☐ 2
- Neither dissatisfied nor satisfied ☐ 3
- Satisfied ☐ 4
- Very satisfied ☐ 5

43 To all:

In the past 5 years, has a doctor or other health professional asked you about your sex life?

One answer only

- Yes ☐ 1
- No ☐ 2

44 **Have you sought help for a sexuality related issue or problem from any of the following sources in the past 5 years?**

Tick all that apply

- Friend ☐ 01,
- Family member ☐ 02,
- Partner ☐ 03,
- Helpline ☐ 04,
- Health center or clinic ☐ 05,
- Internet websites ☐ 06,
- Internet discussion groups ☐ 07,
- Magazines ☐ 08,
- Self-help books/Information leaflets ☐ 09,
- Self-help groups ☐ 10,
- GP/Family doctor ☐ 11,
- Sexual health/GUM/STI clinic ☐ 12,
- Psychiatrist or psychologist ☐ 13,
- Relationship counsellor ☐ 14,
- Other type of clinic or doctor ☐ 15,
- Have not sought any help ☐ 16.

45 **If you have experienced a sexual issue and NOT sought help, what was the reason(s) for this?**

Tick all that apply

- Embarrassed ☐ 1,
- Ashamed ☐ 2,
- Didn't have time ☐ 3,
- Expected it would clear up on its own ☐ 4,
- Symptoms did not bother me ☐ 5,
- I did not know where to seek help ☐ 6,
- Other reasons ☐ 7.

46 **Have you ever, <u>in your life time,</u> been in a situation in which you did or had to do something sexual against your will or without your consent?**

One answer only

- Never ☐ 1
- Yes, before the age of 18 ☐ 2
- Yes, after the age of 18 ☐ 3
- Yes, both before and after the age of 18 ☐ 4

IF YOU NEVER HAD SEX (ANSWERED NO ON QUESTION 36), EXIT THE QUESTIONNAIRE, AND SEND IT TO IPSOS AS SOON AS POSSIBLE.

THANK YOU VERY MUCH FOR YOUR PARTICIPATION!

FOR ALL WHO HAVE EVER HAD SEX (YES ON QUESTION 36): The next questions are about your sex life. Some questions use the term 'having sex'. By this we mean vaginal, oral, or anal sexual intercourse, masturbation, petting or fondling. Some people go through times when they are not interested in sex or find it difficult to enjoy sexual activities. The questions that follow are about some common difficulties that people experience.

47

FOR ALL WHO HAVE HAD SEX AND ANSWERED YES IN QUESTION 36:

IN EACH LINE: ANSWER QUESTION A (EXPERIENCED) FIRST, AND THEN QUESTION B (HOW DISTRESSED IF EXPERIENCED).

Tick one or two boxes in each line

☆

	A: **In the last year, have you experienced any of the following for a period of 3 months or longer?**		B: **If yes on question A: How much distress did this experience cause you?**				
	Yes	No	No distress	Mild distress	Moderate distress	Severe distress	
• Lacked interest in having sex	1 ☐	2 ☐	1 ☐	2 ☐	3 ☐	4 ☐	1
• Lacked enjoyment in sex	1 ☐	2 ☐	1 ☐	2 ☐	3 ☐	4 ☐	2
• Felt anxious during sex	1 ☐	2 ☐	1 ☐	2 ☐	3 ☐	4 ☐	3
• Felt physical pain as a result of sex	1 ☐	2 ☐	1 ☐	2 ☐	3 ☐	4 ☐	4
• Felt no excitement or arousal during sex	1 ☐	2 ☐	1 ☐	2 ☐	3 ☐	4 ☐	5
• Did not reach a climax (experience an orgasm) or took a long time to reach a climax despite feeling excited/aroused	1 ☐	2 ☐	1 ☐	2 ☐	3 ☐	4 ☐	6
• Reached a climax (experienced an orgasm) more quickly than you would like	1 ☐	2 ☐	1 ☐	2 ☐	3 ☐	4 ☐	7
• **If woman:** Had an uncomfortably dry vagina	1 ☐	2 ☐	1 ☐	2 ☐	3 ☐	4 ☐	8
• **If man:** Had trouble getting or keeping an erection	1 ☐	2 ☐	1 ☐	2 ☐	3 ☐	4 ☐	9

48 **Thinking about your sex life in the last year: All things considered, how satisfied are you with your sexual life?**

One answer only

☆

Completely dissatisfied	Dissatisfied	Neither satisfied nor dissatisfied	Satisfied	Completely satisfied	
1 ☐	2 ☐	3 ☐	4 ☐	5 ☐	1

49 **Compared to 10 years ago, how would you rate your overall enjoyment in sex?**

One answer only

- Much lower ☐ 1
- Somewhat lower ☐ 2
- About the same ☐ 3
- Somewhat higher ☐ 4
- Much higher ☐ 5

50 **Compared to 10 years ago, how would you rate the quality of your orgasm?**

One answer only

- Much lower ☐ 1
- Somewhat lower ☐ 2
- About the same ☐ 3
- Somewhat higher ☐ 4
- Much higher ☐ 5
- I do not experience orgasm ☐ 6

51 **In your lifetime, how would you rate your sexual activity throughout most of your life (until the age of 60)?**

One answer only

- I was sexually very active ☐ 1
- I was moderately sexually active ☐ 2
- Neither active nor inactive ☐ 3
- I was moderately sexually inactive ☐ 4
- I was sexually very inactive ☐ 5

52 **Have you had any sexual activity (sexual intercourse, masturbation, petting or fondling) in the past year?**

One answer only

- Yes ☐ 1
- No ☐ 2

THE FOLLOWING QUESTIONS ASK ABOUT THE FREQUENCY OF YOUR SEXUAL ACTIVITIES DURING THE PAST MONTH.

53 **How many times have you had or attempted sexual intercourse (vaginal, anal or oral sex) during the past month?**

One answer only

- None ☐ 1
- Once in the past month ☐ 2
- 2 or 3 times in the past month ☐ 3
- Once a week ☐ 4
- 2 or 3 times a week ☐ 5
- Once a day ☐ 6
- More than once a day ☐ 7

54 **How often did you masturbate in the past month?**

One answer only

- None ☐ 1
- Once in the past month ☐ 2
- 2 or 3 times in the past month ☐ 3
- Once a week ☐ 4
- 2 or 3 times a week ☐ 5
- Once a day ☐ 6
- More than once a day ☐ 7

55 **Thinking about the past month, have you been worried or concerned by the overall frequency of your sexual activities (sexual intercourse, kissing, fondling and petting and masturbation)?**

One answer only

- Not at all worried or concerned ☐ 1
- A little bit worried or concerned ☐ 2
- Moderately worried or concerned ☐ 3
- Very worried or concerned ☐ 4
- Extremely worried or concerned ☐ 5

56 **Have you ever had sexual intercourse?**

One answer only

- Yes ☐ 1
- No ☐ 2

IF YOU NEVER HAD SEX WITH A PARTNER, EXIT THE QUESTIONNAIRE AND SEND IT TO IPSOS AS SOON AS POSSIBLE.

THANK YOU VERY MUCH FOR YOUR PARTICIPATION!

57 **This question is about the person you had sex with most recently, whether this was quite recently or some while ago. Was the person you had sex with most recently your regular spouse or committed partner?**

One answer only

- Yes ☐ 1
- No ☐ 2
- Do not want to answer ☐ 3

PLEASE COMPLETE THE FOLLOWING QUESTIONS WITH YOUR REGULAR SEX PARTNER OR COMMITTED PARTNER IN MIND.

IF YOU DO NOT HAVE A REGULAR OR COMMITTED PARTNER, SKIP TO QUESTION 63

58 **Thinking about your relationship with your partner, how much do you agree or disagree with the following statement:**

I feel emotionally close to my partner when we have sex together

One answer only

- Agree ☐ 1
- Strongly agree ☐ 2
- Neither agree nor disagree ☐ 3
- Disagree ☐ 4
- Strongly disagree ☐ 5

59 **On a scale of 1 to 7, where 1 means 'very happy' and 7 means 'very unhappy': How happy or unhappy are you with your relationship with your partner, all things considered?**

One answer only

	1 Very happy	2 Quite happy	3 Somewhat happy	4 Neither happy nor unhappy	5 Somewhat unhappy	6 Quite unhappy	7 Very unhappy	
	1	2	3	4	5	6	7	
Tick your answer: ...	☐	☐	☐	☐	☐	☐	☐	1

60 **Please indicate how much you agree with each of the following statements with your regular partner or spouse in mind :**

Tick one box on each line

	Strongly agree 1	Agree 2	Neither agree nor disagree 3	Disagree 4	Strongly disagree 5	
• This person completely accepts me as I am.	☐	☐	☐	☐	☐	1
• I can share my deepest thoughts and feelings with this person.	☐	☐	☐	☐	☐	2
• This person cares deeply for me.	☐	☐	☐	☐	☐	3
• This person would willingly help me in any way.	☐	☐	☐	☐	☐	4
• My thoughts and feelings are understood and affirmed by this person.	☐	☐	☐	☐	☐	5
• My partner has experienced sexual difficulties in the last year	☐	☐	☐	☐	☐	6
• My partner and I share about the same level of interest in having sex	☐	☐	☐	☐	☐	7
• My partner and I share the same sexual likes and dislikes	☐	☐	☐	☐	☐	8
• I am not interested in sex	☐	☐	☐	☐	☐	9
• My partner has no interest in sex	☐	☐	☐	☐	☐	10

61 **My partner and I kiss and cuddle each other..........**

One answer only. Tick what fits best.

- Very seldom or seldom ☐ 1
- Often or very often ☐ 2

62 **Over the past 4 weeks , how often have you been sexually touched and caressed by your partner?**

One answer only.

- Not at all, once or twice or weekly ☐ 1
- 2-3 times a week, almost daily or daily ☐ 2

63 **How often have you avoided sex during the last year?**

One answer only

- Never **(⇒ 65)** ☐ 1
- Rarely ☐ 2
- Sometimes ☐ 3
- Often ☐ 4
- Always or almost always ☐ 5

64 **If you have avoided sex: Why have you avoided having sex?**

Tick all that apply

- Relationship problems ☐ 1,
- Worries about sexually transmitted infections ☐ 2,
- Sexual difficulties ☐ 3,
- Partner's sexual difficulties ☐ 4,
- Body image concerns ☐ 5,
- Health problems ☐ 6,
- I'm not interested in sex anymore ☐ 7,
- Other ☐ 8.

65 **In general, do you and your partner use any lubricants during sexual activity?**

One answer only

- Yes ☐ 1
- No ☐ 2

66 **Have you or your partner ever taken any type of medicine or pills to assist your sexual performance, for example Viagra, Cialis, Levitra or Spedra?**

One answer only

- Yes ☐ 1
- No ☐ 2
- Don't know ☐ 3

Thank you very much for your help and great contribution to scientific research on sexual health and habits. Please put the completed questionnaire in the pre paid envelope and post it to IPSOS as soon as possible.
